# Seasonal dynamics of bulk milk somatic cell count in grazing Norwegian dairy goats

**DOI:** 10.3168/jdsc.2023-0457

**Published:** 2023-11-17

**Authors:** Marit Smistad, Ragnhild Aab⊘e Inglingstad, Siv Skeie

**Affiliations:** 1 TINE SA, R&D Department, Farm Advisory Services, Chr. Magnus Falsens vei 18, 1432 Ås, Norway; 2 Faculty of Chemistry, Biotechnology and Food Science, Norwegian University of Life Sciences, N-1432 Ås, Norway

## Abstract

Somatic cell count (SCC) is a widely used indicator of milk quality in the dairy industry. It is a relatively good indicator of udder health in dairy cows, but strongly confounded by non-infection factors, including season, in dairy goats. This study's objectives were to estimate the variation in SCC explained by season and stage of lactation. We also investigated associations between SCC and other milk quality parameters routinely measured, including total bacterial count, fat, protein, and lactose content, in different seasons and stages of lactation. In this observational study, we included results from the routine analyses of bulk milk samples (n = 5,180) collected every third day in 88 Norwegian goat herds through one year. Herd information was collected via a questionnaire and from the dairy herd recording system. The herds had a notable increase in bulk SCC associated with the pasture season. The median bulk SCC was 470,000 cells/mL in deliveries from the indoor spring period and 1,100,000 cells/mL in the pasture season. During the indoor fall season, when most goat herds are in late lactation, the median SCC was 940,000 cells/mL. The combined effect of the season and herd stage of lactation explained 53.5% of the variation in bulk milk SCC. Fat and protein contents varied significantly with season and stage of lactation, and the association with SCC was non-significant for fat content and explained less than 3% of the variation in protein content. Lactose content was associated with SCC, stage of lactation, and season. Total bacterial count was associated with SCC and the indoor seasons. Determination of normal seasonal variation of goat bulk milk SCC is necessary to establish thresholds for defining milk as abnormal and unacceptable for further processing and improving the usefulness of SCC as a tool for milk quality improvement in dairy goats. The results suggest that SCC thresholds for taking action to improve the milk quality should be adjusted for season and stage of lactation.

## Graphical Abstract

**Figure:**
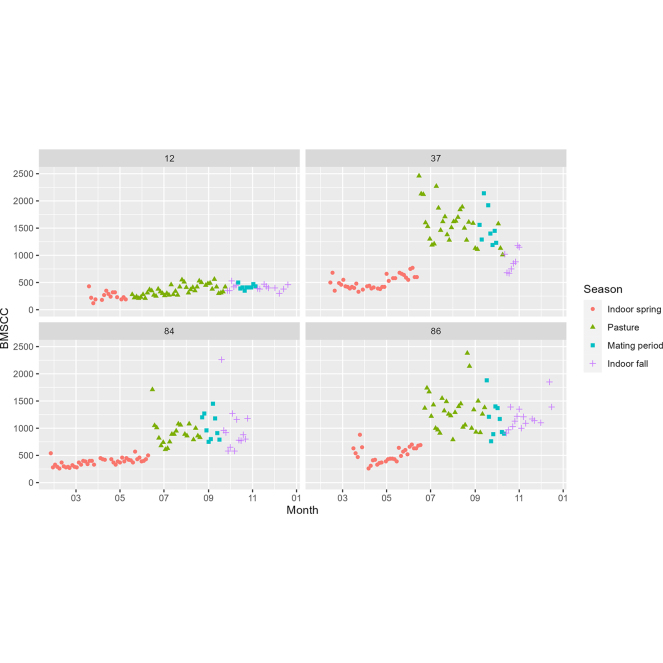

Somatic cell count is an indicator of mastitis and an important measure of milk quality in the dairy industry. Elevated SCC is associated with changes in milk composition, including lactose, fat, and casein contents and total bacterial count (Lindmark-Månsson et al., 2006; Koop et al., 2009; Podhorecká et al., 2021). To encourage the delivery of milk from healthy animals and avoid a detrimental effect on product quality, SCC is often included as a payment parameter by dairy companies.

Goat milk, in general, has considerably higher SCC than cow milk (Paape et al., 2007; Souza et al., 2012). The reasons for this are only partly known. Goat milk has a higher number of neutrophils (Paape and Capuco, 1997; Boutinaud and Jammes, 2002) and non-leukocytic cells, including epithelial cells (Park and Humphrey, 1986; Bagnicka et al., 2011). Due to apocrine milk secretion, goat milk contains apocrine particles, some of which are nucleated and are therefore counted as cells with modern methods (Paape and Capuco, 1997; Bagnicka et al., 2011). Finally, although the bulk milk SCC (**BMSCC**) in goats is related to herd udder health (Koop et al., 2009), it is also significantly influenced by non-infectious factors, including stage of lactation, breed, parity, and management factors (Paape et al., 2007; Goetsch et al., 2011; Lianou et al., 2021a).

A seasonal variation in BMSCC has been described in both dairy cows (Harmon, 1994; Green et al., 2006; Olde Riekerink et al., 2007) and goats (Koop et al., 2009; Margatho et al., 2018). This variation is often partly attributed to the late lactation stage of goat herds with a concentrated kidding season during the winter months. Furthermore, the increase in BMSCC during summer has been associated with a higher incidence of intramammary infections following increased exposure to pathogens in the warmer and more humid periods of the year, as well as heat stress (Olde Riekerink et al., 2007). In goat milk production, the seasonal effect varies with the management system (Goetsch et al., 2011), with productions based on indoor feeding and extended lactations having the highest BMSCC in December and the lowest in June (Koop et al., 2009), whereas productions utilizing pasture usually have the highest BMSCC during the pasture season in July and August (Margatho et al., 2018; Smistad et al., 2021; Scano and Caboni, 2022).

The dairy goat population in Norway consists of 256 flocks, with an average flock size of 128 lactating goats, primarily of the breed Norwegian dairy goat (TINE, 2021). Goat milk production in Norway is seasonal, with most farms having concentrated kidding from January to March, and the goats are on pasture between June and August or September. Approximately 35% of farmers move their goats to a mountain summer farm during the pasture period. Milk composition, total bacterial count (**BMTBC**), and BMSCC are analyzed at each bulk milk collection and form the foundation of the payment for goat milk. To account for the increase in BMSCC during the pasture season, the payment for goat milk in Norway is based on a rolling 12-mo geometric mean of all deliveries. The Norwegian Goat Recording System includes recordings of production, milk quality, and herd information, among others. The Norwegian goat population is free from caprine arthritis encephalitis (CAE-virus), caseous lymphadenitis, and paratuberculosis (Nagel-Alne et al., 2014).

Use of pasture is an important goal of the Norwegian goat milk production, but the marked increase in BMSCC associated with the pasture season is a challenge for most farmers. For the goat milk industry, it is a difficult balance between the demand for good milk quality while still encouraging milk production based on rangeland grazing.

Field experience suggests that the pasture season is a major source of variation in BMSCC under Norwegian management conditions. However, the variation between farms is large, and the amount of variation explained by the season is unknown. We hypothesize that the physiological levels of BMSCC in goat herds on pasture may exceed those defined limits for high-quality milk, often set to between 1,000,000 and 1,500,000 cells/mL (Pirisi et al., 2007; Miller and Lu, 2019). To investigate this, the objective of the present study was to estimate the variation explained by season in goat BMSCC for one year in Norwegian dairy goats. The SCC levels that affect milk composition and cheese quality may vary depending on whether the source of elevation of SCC is of infectious or non-infectious origin. A secondary objective was, therefore, to investigate the association between SCC and other quality measures analyzed routinely in the different seasons.

This study investigated associations between BMSCC and season (defined below), as well as correlations between BMSCC and the other routinely measured components of bulk milk (chemical composition and total bacterial count [**TBC**]). The study period was from Jan. 1, 2021, to Dec. 31, 2021. Dates regarding season (e.g., dates for turning the flock on pasture and start of the mating period) were collected via a questionnaire, based on farmers' written records. The questionnaire was created in Microsoft Forms and was sent by e-mail in December 2021 to all farms delivering goat milk to TINE with a registered e-mail address (n = 252). Descriptive data on the included herds (e.g., herd size, average annual milk yield) and results from analysis of bulk milk were retrieved from the Norwegian Goat Recording System.

According to the standard routines used by TINE in 2021, milk was collected every third day, and bulk milk samples obtained at each milk collection were analyzed for fat, protein, and lactose by Fourier-transform infrared spectrometry (Bentley FTS) and SCC by flow cytometry (Bentley FCM; Bentley Instruments Inc., Chaska, MN), and TBC by a BactoCount IBC (Bentley Instruments Inc.). Both SCC and TBC values >9,999 (×1,000 cells/mL) were truncated to 9,999. The TBC result was not reported if the sample was analyzed more than 36 h after milk collection. The instruments were adjusted and calibrated for goat milk.

Farms responding to the questionnaire (n = 103) were eligible for the study. Reasons for exclusion were errors in herd ID (n = 8), fewer than 10 deliveries on pasture (n = 5), or not stating the dates for release of the flock to pasture (n = 2), resulting in a study population of 88 flocks.

Data were analyzed in R (version 4.1.3; R Core Team, 2022) using the packages lme4 (Bates et al., 2015) and SjPlot (Lüdecke, 2023). Based on questionnaire responses on dates for releasing the flock to pasture, end of pasture season, and start of mating period, the BMSCC results from the 88 included flocks were categorized and described according to 4 seasons: (1) indoor (spring), (2) pasture (summer), (3) mating period, and (4) indoor (fall). The end of the mating period was calculated as 21 d after the start of mating period. The validity of the dates collected via questionnaire was assessed by visual inspection of BMSCC plots for each herd, where we accepted a difference of maximum 2 deliveries between the stated date for release to pasture and the observed rise in BMSCC. Bulk milk samples lacking TBC results were excluded. The average stage of lactation of the herd, calculated as days since the first milk delivery after kidding, was categorized as early (less than 100 d), mid (100–180 d) and late (>180 d) lactation.

Associations between lnBMSCC (outcome) and the season and stage of lactation (explanatory variables) were assessed using a mixed linear regression model with herd as random effect. The association between the geometric mean BMSCC during the indoor (spring) period and pasture was evaluated by univariable linear regression.

The associations between lnBMTBC, fat, protein, and lactose (outcomes) and lnBMSCC and season (explanatory variables) were analyzed in 4 mixed regression models, one for each parameter. Herd was included as random effect. The herd average stage of lactation was included as a confounder. The proportion of variance explained by SCC was calculated as the difference in marginal R^2^ in the full model (including SCC) and the reduced model (without SCC).

Mean herd size was 141 goats (range 47–358). The average 280-d milk production was 516 kg (SD 99) for first-parity goats and 706 kg (SD 124) for goats older than first parity. Five farms had autumn kidding (October–November), and the remaining farms had kidding season between December and April. A total of 43% of respondents moved their herd to a summer farm. Geographical distribution and herd data (herd size, milk yield, kidding season) corresponded well with the overall milk goat population in Norway. Hence, the study population was considered representative for Norwegian goat milk production.

The total number of bulk milk samples from the 88 farms was 6,874 (range 46–129 samples per farm). Samples lacking results for BMTBC (n = 1,694) were excluded, resulting in 5,180 samples used in the statistical analyses. The median BMSCC during pasture was more than doubled compared with the indoor spring level, but the other parameters did not show the same seasonal pattern (Table 1). The mean 12-mo geometric mean BMSCC was 798,000 (SD 204) cells/mL, which is approximately the same level as reported in Greece, where most herds have access to pasture (Lianou et al., 2021b), but higher than reported in herds kept indoors in the Netherlands (Koop et al., 2009). Lower BMSCC during the indoor spring season was associated with lower BMSCC during the pasture season (*P* < 0.01).

**Table 1:** Description of milk quality parameters in 5,180 bulk milk samples from 88 Norwegian dairy goat herds related to seasons and stage of lactation

Season	Samples (n)	Stage of lactation^1^	SCC^2^	Fat %^1^	Protein %^1^	Lactose %^1^	Total bacterial count^2^
Indoor (spring)	2,106	56 (43)	470 (350–620)	4.8 (0.6)	3.4 (0.3)	4.7 (0.2)	31 (20–52)
Pasture (summer)	2,090	161 (47)	1,100 (830–1,470)	4.4 (0.4)	3.3 (0.2)	4.4 (0.1)	31 (19–52)
Mating period	529	212 (16)	1,130 (830–1,490)	4.5 (0.4)	3.5 (0.2)	4.3 (0.1)	37 (24–63)
Indoor (fall)	455	243 (84)	940 (700–1,235)	4.5 (0.4)	3.7 (0.3)	4.3 (0.2)	41 (26–74)
Total (all seasons)	5,180	132 (77)	780 (490–1,190)	4.6 (0.6)	3.4 (0.3)	4.5 (0.2)	32 (21–55)

1 Mean (SD).

2 Median (interquartile range) × 1,000 cells/mL.

The model estimates confirm the major influence of season on BMSCC in Norwegian goat milk production (Table 2). Together, season and stage of lactation explained 53.5% of the variation in BMSCC (marginal R^2^), and when adding the herd effect, more than 73.1% (conditional R^2^). The effect of herd includes, for example, differences in management and herd udder health status. One weakness of the study is the concentrated kidding season during winter/spring in the majority of the herds, which leads to a correlation between season and herd stage of lactation (Table 1); these effects are not possible to completely disentangle based on these data. The least squares means (Table 2) provide estimates of the magnitude of the effects; however, some of the effect is probably absorbed by the other predictor in the model.

**Table 2:** The estimated coefficients (β) and 95% CI for associations between ln-transformed bulk SCC with season and stage of lactation, based on 5,180 bulk milk samples from 88 Norwegian dairy goat herds; herd was included as random effect

Predictor	Estimate		LSM	
	β	CI	SCC	CI
(Intercept)	6.05***	5.99–6.11	424	399–450
Season				
Indoor (spring)	Referent			
Pasture (summer)	0.65***	0.62–0.68	812	788–837
Mating period	0.56***	0.51–0.60	742	706–773
Indoor (fall)	0.47***	0.31–0.52	678	578–713
Stage of lactation				
Early (<100 DIM)	Referent			
Mid (100–180 DIM)	0.34***	0.31–0.37	596	578–614
Late (>180 DIM)	0.42***	0.39–0.46	645	626–671

*** *P* < 0.001.

The increase in BMSCC in goat herds associated with pasture season has, to the authors' knowledge, not been described by others. In dairy cows, some seasonal variation is attributed to increased incidence of intramammary infections due to higher temperature and humidity (Olde Riekerink et al., 2007). Herds with lower BMSCC during the indoor spring period had lower BMSCC during the pasture season. The indoor BMSCC is probably related to the udder health of the herd (Koop et al., 2009). Previous work has shown that dairy cows with intramammary infections in the indoor season had a stronger SCC response of pasture turnout (Coulon et al., 1998; Wredle et al., 2014). In this study, the herd prevalence of intramammary infection was unknown, and the proportion of BMSCC explained by intramammary infection should be further investigated. However, a marked increase in BMSCC was seen in most herds in the descriptive plots (examples in plots 37, 84, and 86 in Graphical Abstract), occurring from the first milk delivery after pasture turnout, too soon for intramammary infections to build up in the herds. Further, the lack of a corresponding increase in BMTBC in the same period points toward a largely non-infectious origin of the elevated BMSCC during the pasture season in this study.

Among non-infectious factors, one possible explanation for the great influence of season may be the substantial contrasts under the Norwegian management system: goats have a relatively long indoor feeding period followed by an extensive pasture season. Many goat herds have their grazing season in mountainous areas, which includes long walking distances, rough rangeland, and less supervision. Non-infectious reasons for the increase of SCC on pasture have been proposed as increased movement, involving mechanical stress to the udder (Coulon et al., 1998), dietary factors (Barnouin et al., 1995), and stress (Harmon, 1994, Haenlein, 2002). The questionnaire revealed that goat farmers who had herds with access to outdoor areas outside the grazing season had a less prominent increase of BMSCC when turned out on pasture (results not shown), which indicates that stress may be involved in the elevation of SCC during the pasture season. We hypothesize that goats with constant access to outdoor areas may not show the same excitement upon pasture turnout. Further, this highlights the importance of smooth transitions to reduce the marked increase in BMSCC.

Given the large effects of season and stage of lactation, dairy companies that encourage use of pasture for goat milk production should continue to calculate the 12-mo geometric mean BMSCC, which was done in Norway in the study period (2021). Otherwise, possible consequences may be that farmers keep the goats indoors, or that farmers delay the kidding season until late spring, to avoid having the herd in mid or late lactation on pasture.

Norwegian goat milk production has continuously reduced SCC for the last 10 years (TINE, 2021). The eradication of important chronic diseases and increased focus on udder health improvement in the same period have probably contributed to this. Some farms also manage to have low BMSCC during the pasture season (plot 12, Graphical Abstract), showing that further improvements may be possible. This can be achieved by assessing farm-specific causes of elevated BMSCC, which may be related not only to udder health but also to stress and pasture management.

A secondary aim of this study was to assess the association between components measured in milk and SCC. The model estimates for the association between fat, protein, lactose, and TBC and SCC are presented in Table 3. Protein and TBC had a significant positive association, and lactose a significant negative association with SCC, when correcting for herd average stage of lactation and season. However, the same problem with correlation between lactation stage, season, and SCC was true also for these models, and the effects were not possible to separate in this study. The coefficients for SCC changed markedly (>20%) when removing lactation stage from the models, showing the importance of including stage of lactation as a confounder when assessing the influence of SCC on milk composition in dairy goats. Decreased milk yield in late lactation leads to a concentration effect of both protein content and SCC (Inglingstad et al., 2016; Eknæs et al., 2017), whereas lactose follows the curve of milk yield, and its concentration is lowest in late lactation (Costa et al., 2019). For fat, protein, and TBC, most of the variation was explained at herd level, and a low proportion of variance (<3%) was explained by SCC.

**Table 3:** The estimated coefficients (β) and 95% CI for associations between content of fat, protein, lactose, and total bacterial count (TBC, ln-transformed) with lnSCC, lactation stage, and season, based on 5,180 bulk milk samples from 88 Norwegian dairy goat herds; herd was included as random effect

Predictor	Fat % β (CI)	Protein % β (CI)	Lactose % β (CI)	lnTBC β (CI)
(Intercept)	4.78 (4.55 to 5.00)***	3.20 (3.09 to 3.31)***	5.43 (5.38 to 5.48)***	1.99 (1.66 to 2.33)***
lnSCC	0.01 (−0.02 to 0.05)^NS^	0.04 (0.02 to 0.05)***	−0.11 (−0.12 to −0.11)***	0.26 (0.20 to 0.31)***
Lactation stage				
Early (<100 DIM)	Referent	Referent	Referent	Referent
Mid (100–180 DIM)	−0.30 (−0.34 to −0.26)***	−0.04 (−0.06 to −0.02)**	−0.17 (−0.18 to −0.16)***	−0.14 (−0.20 to −0.08)***
Late (>180 DIM)	−0.13 (−0.18 to −0.08)***	0.17 (0.14 to 0.19)***	−0.27 (−0.28 to −0.26)***	0.08 (0.00 to 0.15)*
Season				
Indoor (spring)	Referent	Referent	Referent	Referent
Pasture (summer)	−0.24 (−0.29 to −0.20)***	−0.13 (−0.15 to −0.11)***	−0.05 (−0.06 to −0.04)***	−0.17 (−0.24 to −0.10)***
Mating period	−0.28 (−0.34 to −0.22)***	−0.09 (−0.12 to −0.06)***	−0.06 (−0.08 to −0.05)***	−0.15 (−0.24 to −0.06)**
Indoor (fall)	−0.23 (−0.29 to −0.17)***	0.09 (0.05 to 0.12)***	−0.10 (−0.12 to −0.09)***	−0.14 (−0.23 to −0.05)**

* *P* < 0.05

** *P* < 0.01

*** *P* < 0.001; NS = not significant, *P* > 0.05.

The negative association between SCC and lactose has also been shown previously (e.g., Rajčević et al., 2003; Costa et al., 2019; Podhorecká et al., 2021), and is often explained by impaired lactose synthesis due to damage to the milk epithelial cells caused by inflammation. Moreover, lactose is lost to blood due to disruption of the tight junctions between and increased permeability of the basal membrane of mammary epithelial cells (Costa et al., 2019).

The co-variation between SCC and TBC has been hypothesized to be associated with intramammary infections, which may increase both parameters (Koop et al., 2009; Kaskous et al., 2023). In our study, TBC was associated with the indoor seasons (Table 3), which may suggest that BMSCC is a better predictor of herd udder health when the herd is housed.

This study showed that factors other than SCC, including lactation stage and unmeasured herd-level factors, explained more of the variation in other routinely measured milk components. The association between SCC and cheese-making properties of goat milk is unclear. Leitner et al. (2016) found that BMSCC up to a threshold of 3,500,000 cells/mL did not affect the coagulation properties of goat milk. Further, Chen et al. (2010) found no differences in cheese yield when producing cheese from goat milk with low (<500,000), medium (500,000–1,000,000), and high SCC (1,000,000–1,500,000 cells/mL). However, they observed that SCC affected the texture and sensory properties of the cheese, and the cheese made from milk with high SCC had inferior quality. Closer attention to the influence of different cell types and endogenous enzymes on milk and milk coagulation properties, as also underlined by Podhorecká et al. (2021), is important for assessing the importance of the increased SCC during pasture in dairy payment systems.

This study describes the variation of BMSCC through one year in 88 goat herds that were on pasture during the summer months. Goat milk production utilizing non-cultivable land is important for the industry's sustainability and is positive for animal welfare. Pasture-based milk production is also an increasing demand from the consumers of dairy products. Because BMSCC is one of the primary herd udder health indicators in the dairy industry, knowledge of the physiological, management-related variation of BMSCC is essential to recognize a potential herd udder health problem. The study shows that action thresholds should be adjusted for pasture status and stage of lactation.
